# The Effects of Delayed Visual Feedback on Dynamic Postural Control

**DOI:** 10.1167/iovs.66.6.68

**Published:** 2025-06-23

**Authors:** Nora Pourhashemi, Kayton Jaksic, Behrang Keshavarz, Taylor W. Cleworth

**Affiliations:** 1 School of Kinesiology and Health Sciences, York University, Toronto, Ontario, Canada; 2 Centre for Vision Research, York University, Toronto, Ontario, Canada; 3 KITE-Toronto Rehabilitation Institute, University Health Network, Toronto, Ontario, Canada; 4 Department of Psychology, Toronto Metropolitan University, Toronto, Ontario, Canada

**Keywords:** balance, vision, delay, virtual reality, motion sickness

## Abstract

**Purpose:**

Vision provides essential sensory feedback to maintain upright stance yet is affected by inherent processing delays within the central nervous system. Mismatches between visual and motor responses caused by visual delays may also result in motion sickness. In the current study, virtual reality (VR)-generated visual delays were used to examine the relationships among delayed visual feedback, postural responses, and visually induced motion sickness during a dynamic balance task.

**Methods:**

Young healthy adults stood on a force plate mounted to a motorized platform that sinusoidally translated continuously in the anteroposterior (AP) direction for 60 seconds; they wore a VR head-mounted display, surface electromyography (EMG), and full-body motion capture markers. Center of pressure (CoP) was recorded through ground reaction forces using the force plate, kinematics were collected to observe whole-body responses, and surface EMG was used to record muscle activity. Questionnaires were completed after each trial to evaluate subjective measures of perceived stability and visually induced motion sickness.

**Results:**

The amplitude of kinetic, kinematic responses, and muscle activity increased with visual delay and returned to baseline levels when participants were re-exposed to the visual delay conditions.

**Conclusions:**

Strategies used to maintain postural stability under delayed feedback conditions can adapt to sensory delays, without experiencing motion sickness, even if the perceived stability is initially compromised.

The ability to maintain postural stability requires sensory feedback from visual, vestibular, and somatosensory inputs.^1^ Individually manipulating these sensory systems can be used to explore sensory contributions to postural control, as sensory stimuli and/or support surface changes may lead to sensory conflicts.^1^^–^^4^ It has been shown that postural strategies change when vision is altered, where misleading visual inputs lead to increases in sway.^5^ When useful visual information is removed (eyes closed), body sway is increased by 20% to 70%.^1^^,^^6^^,^^7^ Previous work suggests that increasing visual feedback through optic flow gain decreases postural amplitude, especially under challenging conditions (e.g., foam surfaces).^8^ When standing on more challenging conditions, there is an increased reliance on visual cues related to postural control, as larger movements of the head are perceived by the visual system but small movements during quiet stance may not be sufficient to induce visual motion perception.^1^^,^^9^ Standing on compliant surfaces also poses a challenge to the postural control system where proprioceptive input from the feet is reduced, resulting in an increased reliance on visual and vestibular systems to maintain stability.^1^

The shift in sensory reliance underscores the importance of sensory inputs^4^^,^^9^; however, intrinsic delays in the sensorimotor integration may further challenge postural stability.^10^ Physiological processes such as central processing, motor command transmission, and sensory transduction result in delays within the balance control loop, which vary from 80 to 200 ms.^10^^,^^11^ Sensory information from these systems undergoes a brief delay when integrated in the central nervous system to generate motor commands required to maintain upright stance. These delays pose challenges to stability, as longer delays increase postural sway.^10^ Sensorimotor delays have been shown to change over time with aging and disease.^12^^,^^13^ The generation of balance-correcting responses relies on sensory integration and muscle activation, which may create challenges for generalizing these responses across muscle effectors and directions.^14^ This limitation may hinder the ability to accommodate for these delays in postural control, increasing the risk of falling.^14^ Therefore, understanding how one can adapt to visual delays in dynamic stance is crucial, as it provides more translatable learned behavior as compared with quiet stance. However, through training, it has been suggested that healthy adults can overcome these delays,^15^^,^^16^ also known as sensorimotor adaptation. Although aging is associated with increased sensory delays,^15^ the extent to which delays impact postural control remains unclear. Introducing larger visual delays provides a way to systematically probe the adaptability of the postural control system. This also allows for the exploration of visuomotor mechanisms underlying postural control for applications involving extended reality such as virtual reality (VR), where delays may frequently occur.

VR head-mounted displays (HMDs) provide a promising way to alter visual feedback using photorealistic environments,^8^ but they may result in cybersickness.^17^ The inherent delays associated with visual feedback in VR systems is often linked to visually induced motion sickness (VIMS), characterized by symptoms such as nausea, dizziness, and general discomfort.^17^^,^^18^ These delays highlight the importance of improving virtual experience to reduce sickness for VR users. Subjective means to quantify VIMS include the short version of the VIMS Sickness Susceptibility Questionnaire (SSQ),^19^^,^^20^ characterized by a variety of symptoms on different subscales, including oculomotor disturbances, nausea, and disorientation.^18^ Another way to quantify VIMS is by using the Fast Motion Sickness Scale (FMS), a verbal assessment of sickness to quantify motion sickness.^21^ Two of the most prominent theories of motion sickness are sensory conflict^22^^–^^24^ and postural instability.^25^^,^^26^ The sensory conflict theory arises among visual, vestibular, and/or proprioceptive systems if the individual has not established a successful adaptation mechanism. Further, the postural instability theory suggests that motion sickness is associated with changes in body sway, likely when an individual's mechanisms for maintaining stability are impaired. Further work is required to better understand the effects of visual contributions during dynamic postural tasks on motion sickness. By exploring these mechanisms, our understanding of neuromechanical contributions to balance will be improved.

Therefore, the purpose of this study was to examine the effects of delayed visual feedback on postural control and VIMS during support surface translations in young adults. We hypothesized that delayed visual feedback during dynamic stance would result in an increase in postural responses and VIMS responses. We further hypothesized that, during repeated exposure, there would be a reduction in instability quantified through postural responses and VIMS, which may suggest adaptability.

## Methods

### Participants

Twenty healthy (self-reported) adults between the ages of 18 and 40 years old (mean age, 20.2 ± 1.76 years) were recruited to participate in this study, equally split between males and females. Participants were excluded if they reported any neurological, musculoskeletal, orthopedic, and/or prescription medications that may impair their balance. All participants were provided with informed consent prior to participating in the study, in accordance with the Human Participants Review Sub-Committee of York University's Ethics Review Board prior to participation.

### Experimental Procedure and Setup

**Figure 1.**
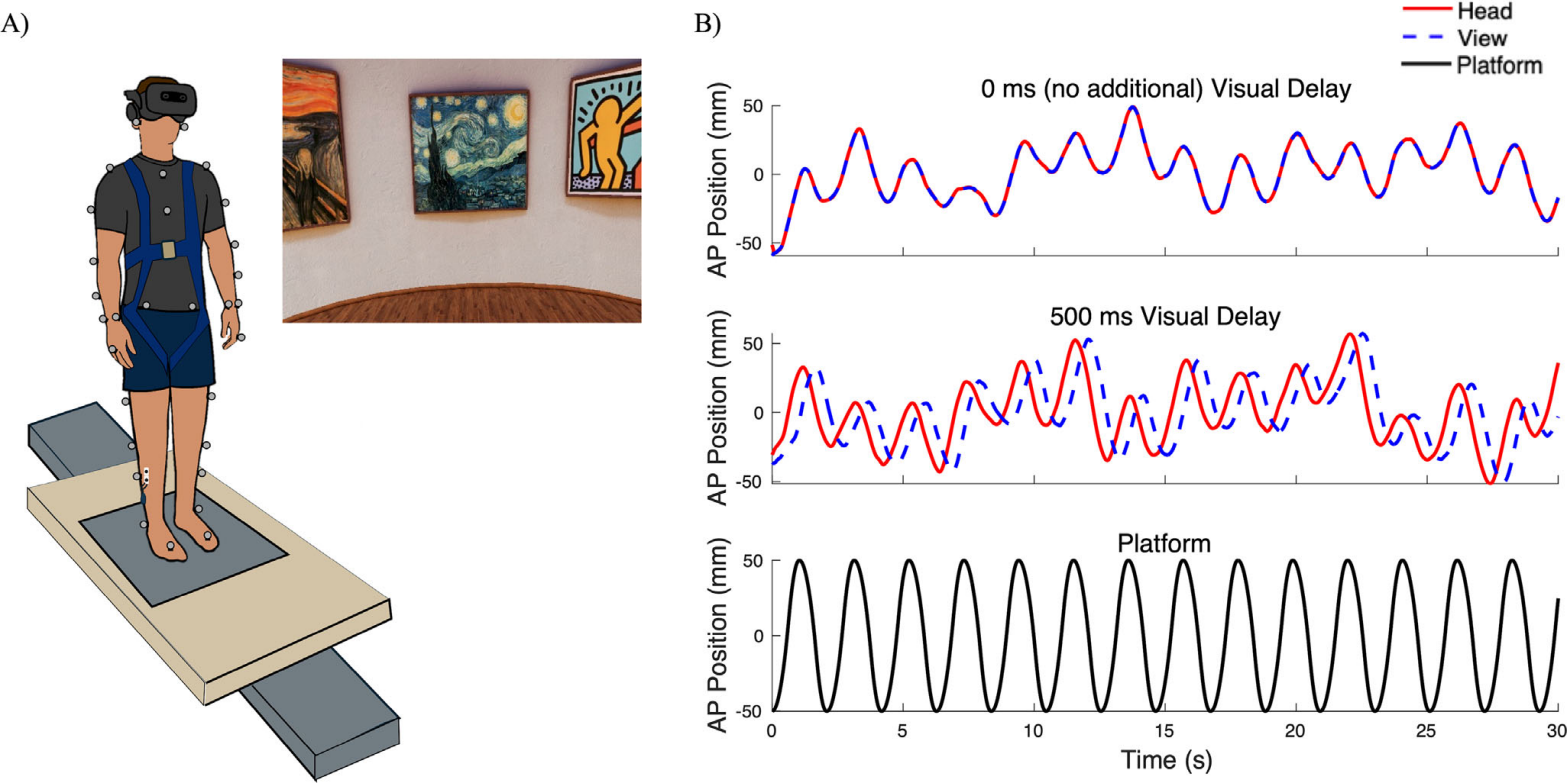
(**A**) Experimental setup: Participants stood on a force plate mounted to a translating platform (1.6 m long **×** 0.9 m wide **×** 0.27 m high) while wearing a VR HMD displaying a virtual scene. EMG and kinematic markers are not illustrated. (**B**) Absolute head position of a representative subject across 30 seconds, with visual delay values of 0 ms and 500 ms and displacement of the translating platform, which continuously moved in the AP direction within a range of ±50 mm.

Participants stood on a force plate (AMTI, Watertown, MA, USA) mounted to a motorized platform for 60 seconds during continuous and sinusoidal translations in the anteroposterior (AP) direction while wearing a VR HMD (Vive Pro 2, 120° horizontal field of view; HTC Corporation, Taoyuan City, Taiwan) with their hands resting naturally by their sides and facing in the direction of the perturbation (Fig. 1). Participants were fitted with a harness that provided enough slack to ensure that no tactile feedback was provided during experimental trials. Stance width was standardized to foot length and marked on the force plate. The support surface perturbation was a continuous translation that moved at 0.5 Hz and translated within a range of ±50 mm from the center of the platform, with a maximum velocity of 0.3 m/s and maximum acceleration of 0.3 m/s^2^. Previous work has reported that, at lower frequencies with a maximum of 0.5 Hz, individuals tended to move with the platform, relying on vision for stabilization.^27^ Beyond 0.5 Hz, postural strategies shift to a multisegmented responses, increasing reliance on vestibular and proprioceptive inputs.^27^ Surface electromyography (EMG) was collected from pairs of surface electrodes placed 2 cm apart along the muscle bellies of the right medial gastrocnemius (MG), tibialis anterior (TA), and soleus (SOL) (Ultium; Noraxon, Scottsdale, AZ, USA). Maximum voluntary contractions (MVCs) were completed for each of the three muscles. Participants completed plantarflexion under resistance while lying prone for the MG, dorsiflexion under resistance for the TA, and plantarflexion under resistance with an approximate 90° knee angle for the SOL.

**Figure 2.**
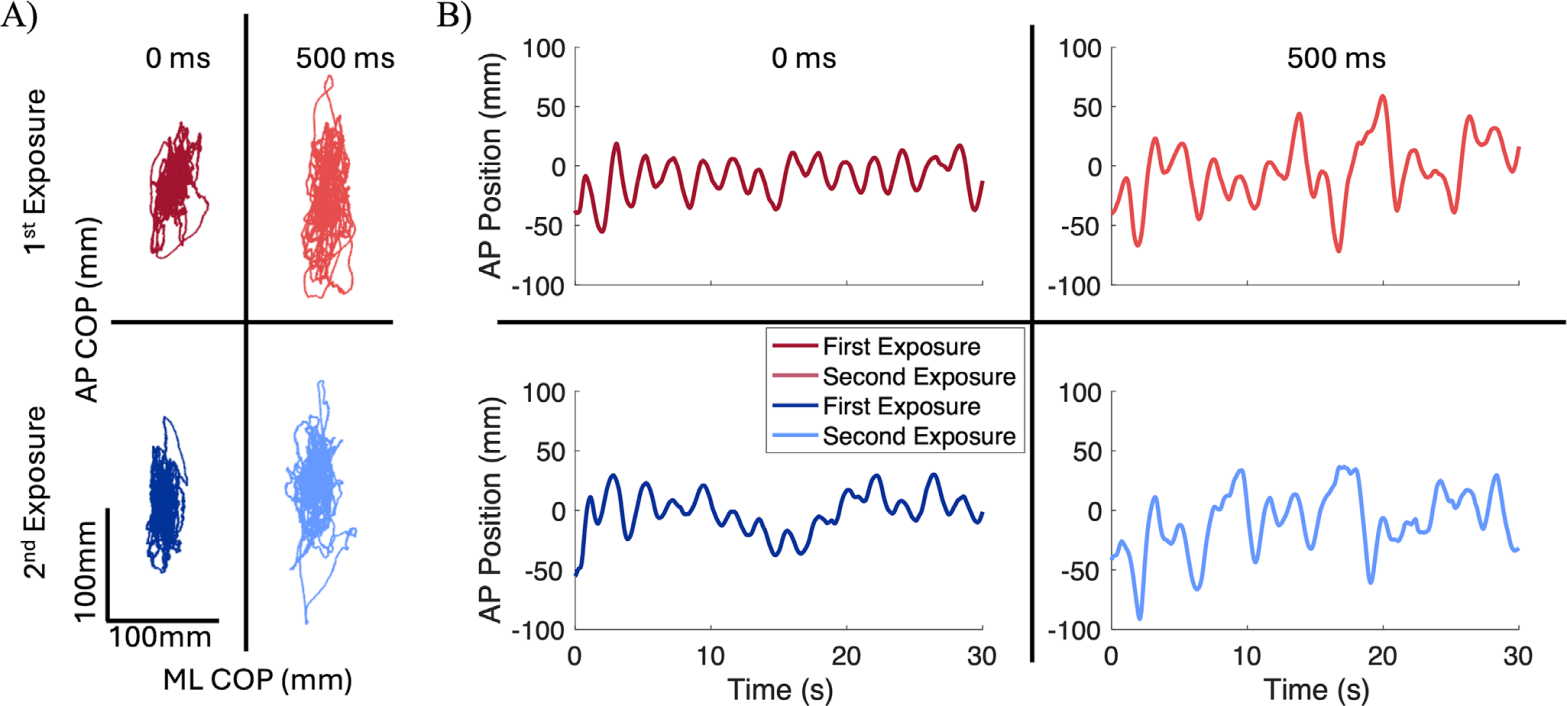
(**A**) Representative spaghetti plots for AP-mediolateral (ML) CoP displacements (mm) and (**B**) traces for AP head position (mm) during baseline (0 ms) and 500 ms delay conditions between exposures.

Participants wore 40 reflective markers placed on the following anatomical landmarks, adapted from the full-body Nexus 2 Plug-In Gait model (Vicon, Centennial, CO, USA): head (on the zygomatic bone and back of the head), glenohumeral joint, upper arm, lateral epicondyle, forearm, radial and ulnar styloid, third metacarpal, anterior superior iliac spine (ASIS), posterior superior iliac spine (PSIS), upper thigh, tibiofemoral joint, tibialis anterior, lateral malleolus, third metatarsal, calcaneus, clavicle, sternum, seventh cervical vertebra (C7), tenth thoracic vertebra (T10), first sacral vertebra, and right upper back. Four markers were placed at each corner of the translating platform to track platform position. The current analysis focused on the head, trunk, and platform markers.

A photorealistic art gallery environment (gallery.osgb, developed by WorldViz, Santa Barbara, CA, USA) was used in all VR conditions where the VR scene dynamically changed based on the movement of the participants head motion. Although inherent delays were not recorded in the current study, previous studies have reported latencies of approximately 22 ms during normal function.^28^ In two experimental conditions, an additional visual delay was manipulated using Vizard python programming by applying time delays of 250 ms and 500 ms relative to head position (Fig. 1B). Specifically, the delay was added using a filter delay function in Vizard, which introduces a temporal delay for a specified time. Participants were exposed to a minimum of three practice trials, lasting 30 seconds each: (1) observing the platform movement, (2) standing on the translating platform without the VR HMD, and (3) standing on the translating platform while wearing the VR HMD, immersed in the virtual environment without visual delay. Prior to the experimental trials, participants were explicitly informed that a visual delay would be introduced in specific conditions. They were given the opportunity to explore the virtual environment before the platform translation commenced. During experimental trials, randomized conditions of the delayed visual feedback were 0 ms (no additional delay), 250 ms, and 500 ms. Each visual delay condition lasted 60 seconds and was repeated once, making a total of two trials per delay condition. After each trial, subjective measures of perceived stability and VIMS were assessed by providing participants with a series of questionnaires. Specifically, perceived stability was assessed from 0% to 100% (0% = “I did not feel stable at all”; 100% = “I felt completely stable”). VIMS was examined through two questionnaires: FMS (0 = no sickness at all, 20 = frank sickness)^21^ and SSQ to assess the severity of motion sickness symptoms on a four-point scale (0 = none, 1 = slight, 2 = moderate, 3 = severe).^29^^,^^30^ These symptoms were categorized into three subscales: nausea, oculomotor, and disorientation, and each subscale was calculated by summing the symptom ratings into specific weights: nausea (9.54), oculomotor (7.58), and disorientation (13.92). The total score was then derived by summing the weighted subscale scores and applying an additional weight of 3.74.^29^^,^^30^ Overall, higher total scores indicated a greater severity of simulator sickness symptoms.^29^^,^^30^ After participants completed the questionnaires, a mandatory rest period of 2 minutes was provided, without standing on the translating platform and without wearing the VR HMD.

### Measures

Ground reaction forces and moments were recorded from a force plate, sampled at 1000 Hz. The center of pressure (CoP) was calculated in MATLAB R2022b (MathWorks, Natick, MA, USA) and was lowpass filtered using a 5-Hz second-order, dual-pass Butterworth filter; bias was removed by subtracting the mean CoP position from the signal. Kinematic data were sampled at 100 Hz and captured using motion capture (Nexus 2 Plug-In Gait). Kinematic data were filtered using a 5-Hz lowpass Butterworth filter, and bias was removed by subtracting the mean position from the signal in MATLAB R2022b. AP CoP, head (using one of four head markers), and trunk (calculated as the average of left and right shoulder markers) root mean square (RMS) values, a measure of amplitude, were calculated using the following formula:

$$\begin{array}{c} RMS=\sqrt{\frac{1}{n}}\sum_{i=1}^{n}x_{i}^{2} \end{array}$$

where *x* is the individual sample and *n* is the number of data points.

EMG was acquired and recorded using the Noraxon Ultium wireless system and digitally recorded in the Nexus 2 Plug-In Gait, sampled at 2000 Hz. EMG data were filtered using a 30- to 500-Hz bandpass filter. Bias was removed, and the data were rectified, normalized to percent MVC, and low-pass filtered at 3 Hz to create a linear envelope. Mean normalized EMG and co-contraction indices (CCIs) TA/SOL and TA/MG were calculated in MATLAB R2022b. CCIs were determined by identifying the point-by-point minimum values between the normalized signals of the agonist and antagonist muscles throughout each trial. These values were subsequently integrated using the trapezoidal rule and normalized by trial duration, yielding the final CCIs.

### Statistical Analysis

Following data collection, a 2 (exposure: first, second) **×** 3 (visual delay: 0 ms, 250 ms, 500 ms) repeated measures ANOVA was used for all outcome measures in SPSS Statistics 29.0, (IBM Corporation, Chicago, IL, USA). Shapiro–Wilks tests and histograms were employed to evaluate normality. Mauchly's test of sphericity was used to assess sphericity, and Greenhouse–Geisser corrections were used if the sphericity assumption was violated. Statistical significance was set at an α-level of 0.05, and Sidak corrections were applied to correct for multiple comparisons. Two participants were excluded from head data analysis, one from trunk data, and three from EMG analyses due to technical errors during data collection. Four of 120 CoP data points (0.03%), seven of 240 head/trunk data points (0.029%), and nine of 600 EMG data points (0.015%) were identified as outliers. Identified outliers were replaced to ±2 SD from the mean.^31^ Outliers were not corrected for questionnaire data.^32^ Significant main effects were explored using multiple paired-sample *t*-tests with a Sidak correction. If normality was violated, a square root transformation was used to correct for normality.

## Results

### Kinetics and Kinematics

**Figure 3.**
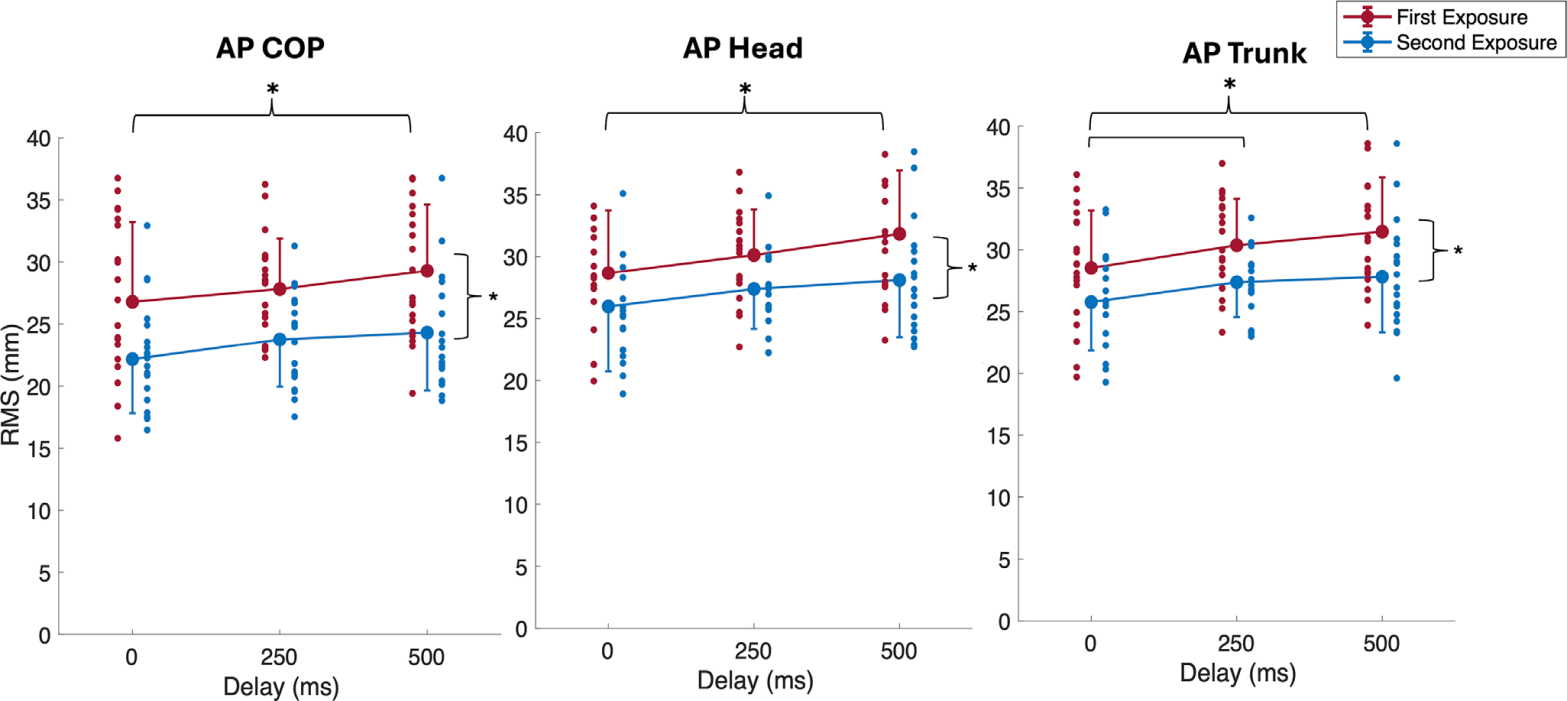
Mean (±1 SD; one-sided error bars) for AP CoP, head, and trunk RMS (mm) across visual delay conditions and exposures. One-sided error bars in the *upward direction* represent mean conditions with greater values, and *downward direction* error bars represent mean conditions with lesser values. *Smaller circles* represent individual participants, and *larger filled circles* are group averages. *Black lines* illustrate post hoc test results for the main effects of visual delay and number of exposures.

During the continuous perturbation, both CoP and body position followed a similar trajectory to the platform; however, increasing the delay had a significant effect on both kinetic and kinematic variables (Figs. 1, 2). There was a significant main effect of delay on AP CoP, trunk, and head RMS (Table 1; Fig. 3), where RMS was greatest at a delay of 500 ms (Table 2). Post hoc results showed that AP CoP and head RMS values had significant changes between the 0-ms and 500-ms delay: for CoP, *t*(19) = 2.786, *P* = 0.035; for head, *t*(17) = 2.804, *P* = 0.036. Post hoc results for AP trunk RMS values showed a significant increase from the 0-ms to 250-ms delay, *t*(18) = 3.164, *P* = 0.016, as well as for the 0-ms to 500-ms delay, *t*(18) = 3.554, *P* = 0.007. There were also significant main effects of exposure for AP CoP, trunk, and head RMS values (Table 1), where RMS decreased during the second exposure (Table 2; Fig. 3). No significant interaction effects were observed for any kinetic or kinematic outcome measures.

**Table 1.** Main Effects From Repeated Measures ANOVA for Kinetics and Kinematics, EMG, and Subjective Outcome Measures

	Exposure			Delay			Exposure*Delay		
	*F* (df1, df2)	*P*	η^2^	*F*( df1, df2)	*P*	η^2^	*F* (df1, df2)	*P*	η^2^
CoP and Body									
AP CoP RMS	**72.427 (1, 19)**	**<0.001**	**0.792**	**4.540 (2, 19)**	**0.017**	**0.193**	0.256 (2, 19)	0.775	0.013
AP trunk RMS	**57.188 (1, 18)**	**<0.001**	**0.761**	**7.525 (2, 18)**	**0.02**	**0.295**	0.344 (1.55, 18)	0.658	0.019
AP head RMS	**30.014 (1, 17)**	**<0.001**	**0.638**	**4.415 (2, 17)**	**0.02**	**0.206**	0.428 (2, 17)	0.655	0.025
EMG									
% MVC TA	**17.060 (1, 15)**	**<0.001**	**0.532**	**5.294 (2, 15)**	**0.011**	**0.261**	2.764 (2, 15)	0.79	0.156
% MVC SOL	**0.759 (1, 17)**	**0.014**	**0.309**	1.866 (1.33, 17)	0.185	0.099	0.927 (2, 17)	0.406	0.052
% MVC MG	**12.195 (1, 17)**	**0.003**	**0.418**	2.003 (1.46, 17)	0.165	0.105	0.303 (2, 17)	0.741	0.017
CCI TA/SOL	**12.999 (1, 15)**	**0.003**	**0.464**	**4.383 (2, 15)**	**0.021**	**0.226**	2.149 (2, 15)	0.134	0.125
CCI TA/MG	**19.728 (1, 16)**	**<0.001**	**0.552**	2.822 (2, 16)	0.074	0.15	2.390 (2, 16)	0.108	0.13
Subjective									
Stability	0.004 (1, 19)	0.952	0.00	**7.046 (2, 19)**	**0.002**	**0.271**	0.338 (2, 19)	0.716	0.017
FMS	0.09 (1, 19)	0.767	0.005	1.878 (2, 19)	0.18	0.09	0.452 (2, 19)	0.639	0.023
SSQ total	0.708 (1, 19)	0.411	0.036	1.771 (2, 19)	0.197	0.085	1.597 (2, 19)	0.216	0.078

Bolded values denote statistical significance.

**Table 2.** Means and 95% CIs Across Kinetics and Kinematics, EMG, and Subjective Outcome Measures

	First Exposure, Mean (SD) [95% CI]			Second Exposure, Mean (SD) [95% CI]		
	0 ms	250 ms	500 ms	0 ms	250 ms	500 ms
CoP and Body						
AP CoP RMS	26.78 (6.43) [23.77–29.78]	27.80 (4.08) [25.90–29.71]	29.28 (5.35) [26.78–31.78]	22.16 (4.33) [20.14–24.20]	23.75 (3.79) [21.97–25.52]	24.31 (4.65) [22.13–26.48]
AP trunk RMS	28.53 (4.67) [26.27–30.78]	30.37 (3.78) [28.54–32.18]	31.46 (4.38) [29.35–33.57]	25.76 (3.91) [23.87–27.64]	27.36 (2.82) [26.00–28.72]	27.81 (4.49) [25.65–29.97]
AP head RMS	28.66 (5.08) [26.13–31.19]	30.12 (3.68) [28.29–31.95]	31.83 (5.11) [29.28–34.37]	25.96 (5.24) [23.35–28.57]	27.37 (3.22) [25.77–28.97]	28.08 (4.60) [25.80–30.37]
EMG						
% MVC TA	0.83 (0.30) [0.67–0.99]	0.99 (0.39) [0.78–1.20]	1.00 (0.38) [0.80–1.21]	0.77 (0.28) [0.63–0.92]	0.82 (0.29) [0.67–0.97]	0.85 (0.33) [0.68–1.03]
% MVC SOL	1.27 (0.85) [0.85–1.69]	1.50 (2.13) [0.94–2.07]	1.45 (0.98) [0.96–1.94]	1.18 (0.91) [0.72–1.63]	1.22 (0.79) [0.83–1.61]	1.25 (0.91) [0.80–1.70]
% MVC MG	1.06 (0.38) [0.87–1.25]	1.14 (0.47) [0.91–1.37]	1.13 (0.42) [0.92–1.34]	1.00 (0.42) [0.80–1.21]	1.04 (0.38) [0.85–1.23]	1.04 (0.42) [0.84–1.25]
CCI TA/SOL	0.63 (0.23) [0.51–0.75]	0.71 (0.24) [0.58–0.84]	0.73 (0.24) [0.60–0.85]	0.58 (0.22) [0.46–0.70]	0.64 (0.25) [0.50–0.77]	0.62 (0.21) [0.51–0.73]
CCI TA/MG	0.64 (0.23) [0.52–0.76]	0.72 (0.21) [0.61–0.83]	0.72 (0.19) [0.62–0.82]	0.60 (0.21) [0.49–0.70]	0.62 (0.19) [0.52–0.71]	0.63 (0.19) [0.53–0.72]
Subjective						
Stability	85.35 (15.78) [77.96–92.74]	79.85 (20.18) [70.41–89.29]	79.90 (14.10) [73.30–86.50]	85.20 (17.10) [77.21–93.20]	81.05 (16.83) [73.17–88.93]	79.25 (18.70) [70.51–87.99]
FMS	1.20 (2.61) [−0.02–2.42]	2.10 (3.08) [0.66–3.54]	2.40 (4.10) [0.48–4.32]	1.40 (2.52) [0.22–2.58]	1.95 (3.20) [0.46–3.44]	1.90 (3.04) [0.48–3.32]
SSQ total	6.36 (9.79) [1.78–10.94]	6.36 (7.59) [2.81–9.91]	10.66 (12.86) [4.64–16.68]	8.60 (13.02) [2.51–14.69]	10.10 (14.77) [3.19–17.00]	10.66 (14.27) [3.98–17.34]

### EMG Data

**Figure 4.**
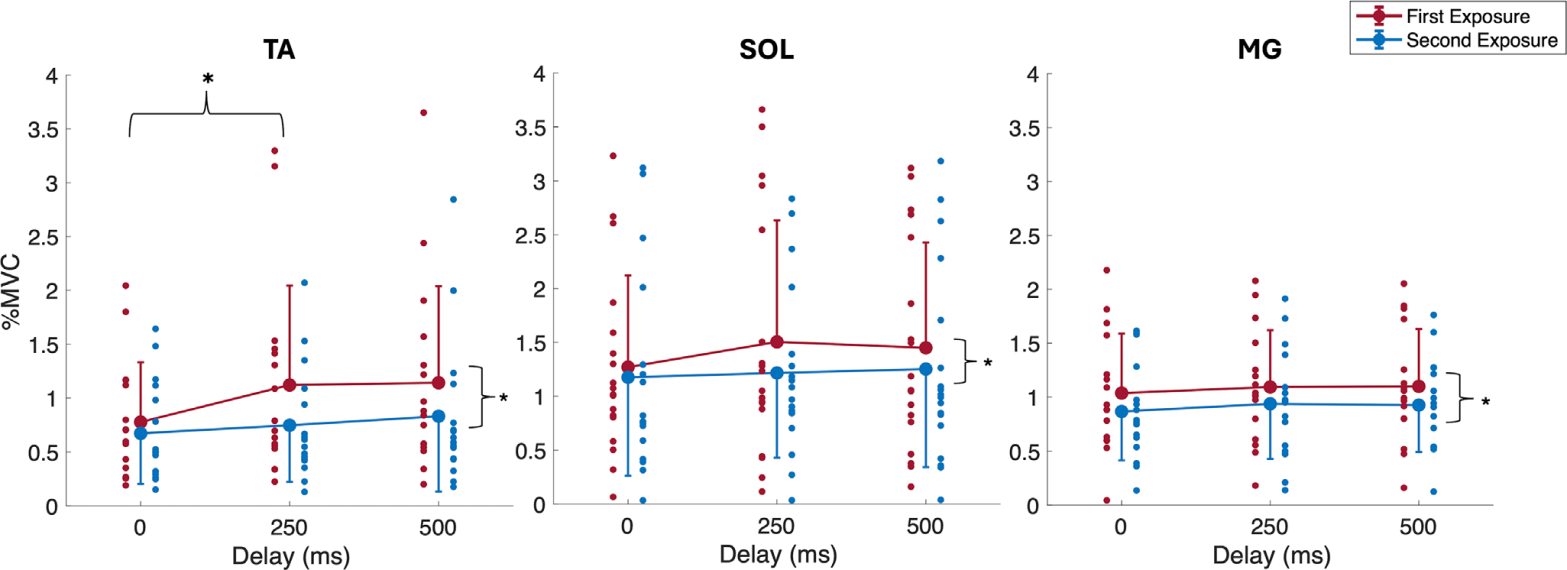
Mean (±1 SD; one-sided error bars) percent MVC activation for TA, SOL, and MG muscles across visual delay conditions and exposures. One-sided error bars in the *upward direction* represent mean conditions with greater values, and *downward direction* error bars represent mean conditions with lesser values. *Smaller circles* represent individual participants, and *larger filled circles* are group averages. *Black lines* illustrate post hoc test results for the main effects of visual delay and number of exposures.

**Figure 5.**
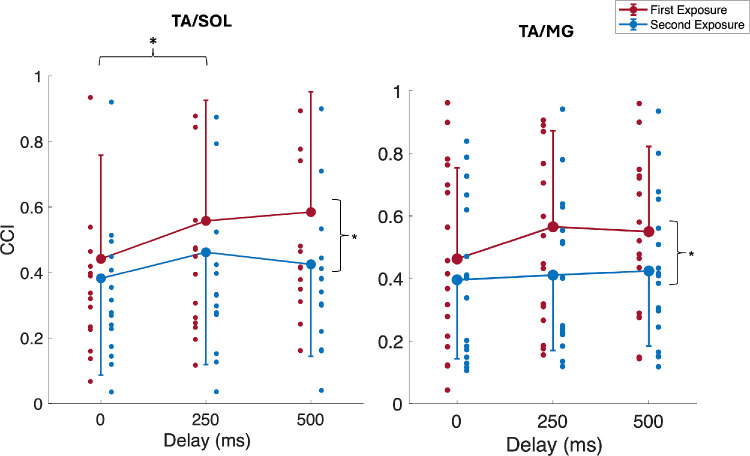
Mean (±1 SD; one-sided error bars) for CCIs of TA/SOL and TA/MG across visual delay conditions and exposures. One-sided error bars in the *upward direction* represent mean conditions with greater values, and *downward direction* error bars represent mean conditions with lesser values. *Smaller circles* represent individual participants, and *larger filled circles* are group averages. *Black lines* illustrate post hoc test results for the main effects of visual delay and number of exposures.

There were significant main effects of delay for TA activity (Table 1), where post hoc analyses showed a significant increase between the 0-ms and 250-ms delay, *t*(15) = 3.571, *P* = 0.008. No significant main effects of delay were observed for SOL or MG activity (Table 1). In addition, the CCI for TA/SOL significantly increased with visual delay (Table 2). Post hoc analyses showed a significant increase in TA/SOL CCIs from the 0-ms to 250-ms delay, *t*(15) = 3; *P* = 0.029. There were also significant main effects of exposure for all muscle activity responses (Table 1), such that the activity of all three muscles and CCIs decreased during the second exposure (Figs. 4, 5). No significant main effects of delay were shown for the CCI for TA/MG. No significant interaction effects were observed for any EMG outcome measure.

### Subjective Measures

**Figure 6.**
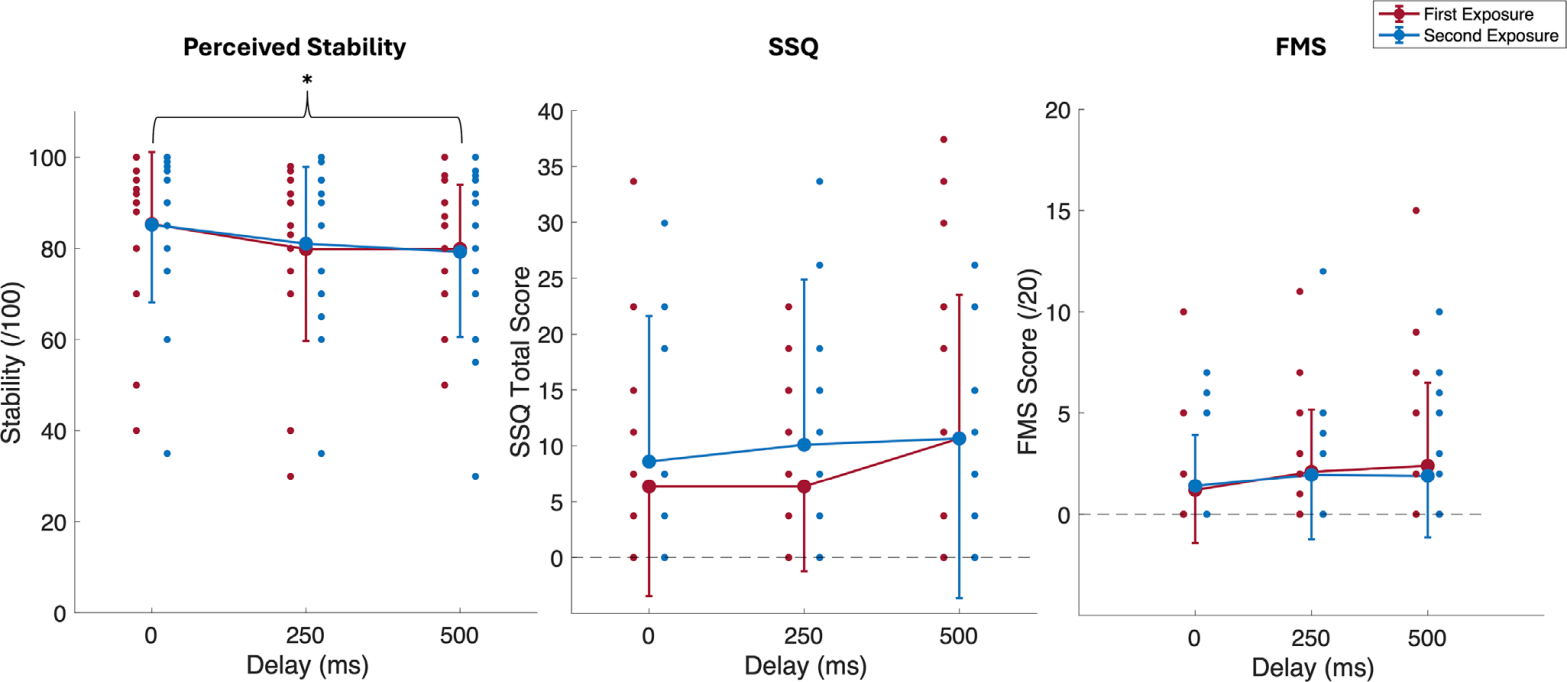
Mean scores (±1 SD; one-sided error bars) for perceived stability, SSQ, and FMS across visual delay and exposures. One-sided error bars in the *upward direction* represent mean conditions with greater values, and *downward direction* error bars represent mean conditions with lesser values. *Smaller circles* represent individual participants, and *larger filled circles* are group averages. *Black lines* illustrate post hoc test results for the main effects of visual delay and number of exposures.

There was a significant main effect of delay on perceived stability (Table 1; Fig. 6). Post hoc analyses showed a significant decrease in perceived stability from the 0-ms to 500-ms delay, *t*(19) = 3.53, *P* = 0.007. No significant main effects of exposure for perceived stability or main effects of delay or exposure were observed for either motion sickness measure (FMS or SSQ) (Table 1). No significant interaction effects were observed for all subjective measures.

## Discussion

The aim of this study was to examine the effects of delayed visual feedback on dynamic postural control and VIMS in young adults. At first exposure, participants showed large postural and subjective responses with delayed visual feedback, supporting the importance of reliable visual input in maintaining balance. Upon second exposure, the postural responses were significantly reduced. The postural control system was able to adapt to visual delays, which may be explained by various concepts including sensory reweighting, a decrease in sensorimotor gain, and/or utilizing the delayed visual feedback. Our findings align with early work demonstrating the dominant influence of vision in postural control, particularly when visual input conflicts with vestibular and proprioceptive systems.^33^ We expanded this work by demonstrating that, although vision remains a strong driver of postural control, its influence under delayed visual feedback conditions does not diminish within a single trial. According to Bronstein (2019),^34^ when visual cues are presented in conflict with other systems such as vestibular and proprioceptive, the sensory system may downregulate the gain assigned to visual input, shifting its reliance to more reliable systems. This adaptive process may explain the decrease in postural responses over repeated exposures in the present study. One explanation for the variability within individuals may be visual dependence,^33^ as highly visually dependent subjects may be less efficient in downweighing misleading visual information.

Postural responses require complex sensorimotor integration when combined with visual delay. The results in this study further support the idea that, under challenging conditions, the postural control system relies heavily on visual input,^1^ as the amplitude of postural responses increase with increased visual delay, emphasizing the importance of vision in postural control.^5^ Results also showed an increase in muscle activity upon exposure to delay the first time, which suggests a neuromuscular response aimed at countering the visual delay, a strategy applied during unstable conditions.^11^ Previous work suggests that visual delay is processed as an unreliable source of information, resulting in a compensatory increase in muscle activity.^35^ The results of this study found that, even under challenging demands of complex sensorimotor integration, achieved through support surface translations, subjects were still able to adapt to the visual delay. In previous work,^15^ subjects were able to use experience to adapt and learn upon repeated exposure to delay. Muscle activity from TA and TA/SOL co-contraction and trunk amplitude were significantly affected by a delay of 250 ms, demonstrating that 250 ms was sufficient to demonstrate the impact of brief sensorimotor delays on postural control mechanisms.

Postural responses were reduced significantly upon second exposure, suggesting an adaptive response to the visual delay. Previous studies have shown that, following the second exposure to the visual feedback conditions, adaptive changes in the regulation of whole-body responses were learned by the participants.^14^^,^^36^ The nervous system can apply learned control mechanisms by integrating whole-body sensory feedback, overcoming the imposed delay limitations.^15^ A potential sensory reweighting mechanism may be used, where the nervous system adapts to the visual delay by downregulating its reliance on the visual system and increasing the weight of vestibular and somatosensory inputs.^37^ Results from this study further support the theory that flexible and rapid reweighting of sensory information is an important component in our ability to maintain postural stability under challenging conditions,^38^ as participants were able to adapt to the visual conditions effectively. Another proposed mechanism of adaption is the downregulation of sensorimotor gain relative to the increased visual delay. Previous work using computational models on the effects of delayed feedback has demonstrated that adaptive postural responses leading to an increase in postural stability result from a reduction in the amount of neural feedback gain.^39^ Furthermore, the results of this study support existing evidence of adaptive mechanisms in response to delayed visual feedback on postural control, where it was hypothesized that participants reduced the amount of motor action necessary to respond to deviations from their desired position.^14^^,^^40^ A mismatch in internal compared to external delay has been shown to affect feedforward mechanisms, potentially inducing instability.^41^ Foulkes and Miall^42^ examined the effect of delayed visual feedback on tracking performance and proposed two potential adaptive mechanisms based on cerebellar function models, where the open loop gain can be reduced in response to the visual delay or the internal delay can be adapted. They demonstrated that adapting the internal delay was significantly more effective than reducing the open loop gain at increasing performance on a motor task with delayed visual feedback.^41^^,^^42^ Accordingly, another adaptive mechanism that may have been utilized by participants in this study is integration of the visual delay into their internal feedback model, potentially improving motor performance when exposed to visual delay. Future work should examine the changes in the frequency and velocity of the postural responses, as this may yield further insight into the mechanisms of the observed adaptation and whether an open loop or closed loop adaptation mechanism is utilized.^14^

The findings of this study demonstrated a dissociation between sensorimotor adaptation and subjective measures of stability. During the second exposure to increased visual delay, the sway associated with postural responses during the dynamic balance task reduced significantly but a decrease in perceived stability remained, suggesting adaptation in postural behavior and a disconnect between perceived and objective responses. The postural control system was able to adapt to visual delays, which may be explained by sensory reweighting, where there is an adaptation to visual delay conditions but an increased reliance on vestibular and somatosensory systems, as well as a decrease in sensorimotor gain (specifically vision) and/or utilizing the delayed visual feedback. Alternatively, the second exposure results may be related to a shift from automatic to conscious control of movement.^33^ The relationship between adapted postural control strategies to reduce excessive movement and perceived stability is particularly relevant for individuals with idiopathic dizziness^43^ and/or persistent postural–perpetual dizziness (PPPD), a chronic vestibular syndrome characterized by dizziness during upright posture or walking and exposure to dynamic visual stimuli.^44^ In these populations, a decrease in perceived stability is often reported despite undiagnosed or unobserved balance deficits or changes (compared to otherwise healthy controls).^43^ In addition, previous work has suggested that older adults may perceive less self-motion despite greater postural responses compared to younger adults, although the robustness of this finding remains uncertain.^45^ This disconnect between perceived and objective responses may be related to the changes described above in sensorimotor integration mechanisms involved in upright stance. Future work examining perceptions of instability may consider using visual and mechanical perturbations to investigate the relationship between perceived and objective postural responses during dynamic balance tasks. These findings could inform mechanisms of postural control, sensorimotor integration, and visuomotor adaption.

### Delayed Visual Feedback and VIMS

In the present study, we also aimed to investigate relationships between postural responses and severity of sickness. It has been suggested that, within the postural instability theory, motion sickness is likely to occur when the individual's ability to maintain stability is impaired.^29^ Therefore, adaptation to visual delays in postural control may not necessarily imply adaptation in the context of motion sickness, as participants were not affected by VIMS during the first exposure. VIMS remained unaffected despite the increase in visual delay and across exposures, suggesting differences between sensorimotor adaptations in postural control and the mechanisms that typically induce motion sickness. The sensory conflict theory suggests that VIMS occurs if the individual has not established a successful adaptation mechanism.^25^ Previous work has shown that head movement amplitude and display lag influence the severity of cybersickness.^46^ Results of this study suggest that participants may have exhibited smaller amplitudes of head movement, as they did not experience a level of sensory conflict severe enough to trigger motion sickness symptoms. Future work should continue to examine the relationship between postural stability and VIMS by determining thresholds at which visual delay begins to provoke sensory conflict sufficient enough to induce VIMS and explore nonlinear analyses to assess whether certain delays provoke VIMS.

### Limitations

The present study manipulated delayed visual feedback relative to head position using a VR HMD; yet, one limitation is the minimal inherent delay caused by system processing. However, recent evidence suggests that the improvements in VR technology have a minimal (or null) effect on upright stance.^36^ Finally, the head movement associated with dynamic stance may not have been large enough to induce motion sickness, as previous work suggests that head movement amplitude and display lag may influence the severity of sickness.

## Conclusions

Altogether, subjective and objective findings illustrate a complex interaction among visual feedback delays, motor control responses, and individual perceptions of stability and discomfort. Findings from this study indicate that increasing delayed visual feedback resulted in larger postural responses, highlighting the critical role of visual cues, sensory reweighing, and feedback control mechanisms during dynamic balance control. Although perceived stability decreased with visual delay, this effect of delay on perceived stability remained unchanged upon re-exposure. Adaptation mechanisms in this study align with feedback control models, where sensorimotor gains change to maintain postural stability under delayed feedback conditions, suggesting that the body can adapt to sensory delays, without experiencing motion sickness, even if the perceived stability is initially compromised. Understanding how the visual system is employed in dynamic postural control can be applied to studying the role of vision in balance deficits and fall risk interventions while minimizing any adverse side effects of motion sickness. Future research can examine postural control strategies in those who have increased visual reliance such as older adults^47^ and may also incorporate alternative feedback modalities (e.g., auditory or haptic feedback) that can be used to compensate for visual delay.

## References

[1] Lord SR, Menz HB. Visual contributions to postural stability in older adults. *Gerontology*. 2000; 46(6): 306–310. 11044784 10.1159/000022182

[2] Forbes PA, Chen A, Blouin JS. Sensorimotor control of standing balance. *Handb Clin Neurol*. 2018; 159: 61–83. 30482333 10.1016/B978-0-444-63916-5.00004-5

[3] Hwang S, Agada P, Kiemel T, Jeka JJ. Dynamic reweighting of three modalities for sensor fusion. *PLoS One*. 2014; 9(1): e88132. 24498252 10.1371/journal.pone.0088132 PMC3909337

[4] Peterka RJ . Sensorimotor integration in human postural control. *J Neurophysiol*. 2002; 88(3): 1097–1118. 12205132 10.1152/jn.2002.88.3.1097

[5] Lee DN, Lishman JR. Visual proprioceptive control of stance. *J Hum Mov Stud.* 1975; 1: 87–95.

[6] Krishnamoorthy V, Yang JF, Scholz JP. Joint coordination during quiet stance: effects of vision. *Exp Brain Res*. 2005; 164(1): 1–17. 15841397 10.1007/s00221-004-2205-6

[7] Diener HC, Dichgans J, Bruzek W, Selinka H. Stabilization of human posture during induced oscillations of the body. *Exp Brain Res*. 1982; 45(1-2): 126–132. 7056318 10.1007/BF00235771

[8] Lavalle LK, Cleworth TW. The effect of modified optic flow gain on quiet stance. *Neurosci Lett*. 2023; 797: 137068. 36641046 10.1016/j.neulet.2023.137068

[9] Redfern MS, Yardley L, Bronstein AM. Visual influences on balance. *J Anxiety Disord*. 2001; 15(1–2): 81–94. 11388359 10.1016/s0887-6185(00)00043-8

[10] Peterka RJ . Postural control model interpretation of stabilogram diffusion analysis. *Biol Cybern*. 2000; 82(4): 335–343. 10804065 10.1007/s004220050587

[11] Masani K, Vette AH, Kawashima N, Popovic MR. Neuromusculoskeletal torque-generation process has a large destabilizing effect on the control mechanism of quiet standing. *J Neurophysiol*. 2008; 100(3): 1465–1475. 18596181 10.1152/jn.00801.2007

[12] Dorfman LJ, Bosley TM. Age-related changes in peripheral and central nerve conduction in man. *Neurology*. 1979; 29(1): 38–44. 570675 10.1212/wnl.29.1.38

[13] Cameron MH, Nilsagard Y. Balance, gait, and falls in multiple sclerosis. *Handb Clin Neurol*. 2018; 159: 237–250. 30482317 10.1016/B978-0-444-63916-5.00015-X

[14] Rasman BG, Blouin JS, Nasrabadi AM, et al. Learning to stand with sensorimotor delays generalizes across directions and from hand to leg effectors. *Commun Biol**.* 2024; 7: 384. 38553561 10.1038/s42003-024-06029-4 PMC10980713

[15] Rasman BG, Forbes PA, Peters RM, et al. Learning to stand with unexpected sensorimotor delays. *eLife*. 2021; 10: e65085. 34374648 10.7554/eLife.65085 PMC8480973

[16] Rasman BG, van der Zalm C, Forbes PA. Age-related impairments and influence of visual feedback when learning to stand with unexpected sensorimotor delays. *Front Aging Neurosci*. 2023; 15: 1325012. 38161590 10.3389/fnagi.2023.1325012 PMC10757376

[17] Stauffert JP, Niebling F, Latoschik ME. Latency and cybersickness: impact, causes, and measures. A review. *Front Virtual Real*. 2020; 1: 582204.

[18] Kennedy RS, Drexler J, Kennedy RC. Research in visually induced motion sickness. *Appl Ergon*. 2010; 41(4): 494–503. 20170902 10.1016/j.apergo.2009.11.006

[19] Golding JF, Rafiq A, Keshavarz B. Predicting individual susceptibility to visually induced motion sickness by questionnaire. *Front Virtual Real*. 2021; 2: 576871.

[20] Keshavarz B, Murovec B, Mohanathas N, Golding JF. The Visually Induced Motion Sickness Susceptibility Questionnaire (VIMSSQ): estimating individual susceptibility to motion sickness-like symptoms when using visual devices. *Hum Factors*. 2023; 65(1): 107–124. 33874752 10.1177/00187208211008687 PMC9846380

[21] Keshavarz B, Hecht H. Validating an efficient method to quantify motion sickness. *Hum Factors*. 2011; 53(4): 415–426. 21901938 10.1177/0018720811403736

[22] Oman CM . Motion sickness: a synthesis and evaluation of the sensory conflict theory. *Can J Physiol Pharmacol*. 1990; 68(2): 294–303. 2178753 10.1139/y90-044

[23] Reason JT . Motion sickness adaptation: a neural mismatch model. *J R Soc Med*. 1978; 71(11): 819–829. 731645 10.1177/014107687807101109 PMC1436193

[24] Reason JT, Brand JJ. *Motion Sickness*. New York: Academic Press; 1975.

[25] Riccio GE, Stoffregen TA. An ecological theory of motion sickness and postural instability. *Ecol Psychol*. 1991; 3(3): 195–240.

[26] Stoffregen TA, Riccio GE. An ecological critique of the sensory conflict theory of motion sickness. *Ecol Psychol*. 1991; 3: 159–194.

[27] Buchanan JJ, Horak FB. Emergence of postural patterns as a function of vision and translation frequency. *J Neurophysiol*. 1999; 81(5): 2325–2339. 10322069 10.1152/jn.1999.81.5.2325

[28] Niehorster DC, Li L, Lappe M. The accuracy and precision of position and orientation tracking in the HTC Vive virtual reality system for scientific research. *iPerception*. 2017; 8(3): 2041669517708205. 28567271 10.1177/2041669517708205 PMC5439658

[29] Kennedy RS, Lane NE, Berbaum KS, Lilienthal MG. Simulator sickness questionnaire: an enhanced method for quantifying simulator sickness. *Int J Aviat Psychol*. 1993; 3: 203–220.

[30] Golding JF . Predicting individual differences in motion sickness susceptibility by questionnaire. *Pers Individ Dif**.* 2006; 41: 237–248.

[31] Field A . *Discovering Statistics Using SPSS*. 3rd ed. London: SAGE Publications; 2009.

[32] Zijlstra WP, van der Ark LA, Sijtsma K. Outlier detection in test and questionnaire data. *Multivariate Behav Res*. 2007; 42(3): 531–555. 10.1080/00273170701360803 26765492

[33] Bronstein AM . Suppression of visually evoked postural responses. *Exp Brain Res*. 1986; 63(3): 655–658. 3489640 10.1007/BF00237488

[34] Bronstein AM. A conceptual model of the visual control of posture. *Prog Brain Res.* 2019; 248: 285–302. 31239139 10.1016/bs.pbr.2019.04.023

[35] Baweja HS, Patel BK, Martinkewiz JD, Vu J, Christou EA. Removal of visual feedback alters muscle activity and reduces force variability during constant isometric contractions. *Exp Brain Res*. 2009; 197(1): 35–47. 19544059 10.1007/s00221-009-1883-5 PMC3631578

[36] Shiller DM, Veilleux LN, Marois M, Ballaz L, Lemay M. Sensorimotor adaptation of whole-body postural control. *Neuroscience*. 2017; 356: 217–228. 28549560 10.1016/j.neuroscience.2017.05.029

[37] Assländer L, Streuber S. Virtual reality as a tool for balance research: eyes open body sway is reproduced in photo-realistic, but not in abstract virtual scenes. *PLoS One*. 2020; 15(10): e0241479. 33119679 10.1371/journal.pone.0241479 PMC7595375

[38] Ketterer J, Ringhof S, Gehring D, Gollhofer A. Sinusoidal optic flow perturbations reduce transient but not continuous postural stability: a virtual reality-based study. *Front Physiol*. 2022; 13: 803185. 35665227 10.3389/fphys.2022.803185 PMC9157535

[39] Bingham JT, Choi JT, Ting LH. Stability in a frontal plane model of balance requires coupled changes to postural configuration and neural feedback control. *J Neurophysiol*. 2011; 106(1): 437–448. 21543754 10.1152/jn.00010.2011 PMC3129728

[40] van der Kooij H, Peterka RJ. Non-linear stimulus-response behavior of the human stance control system is predicted by optimization of a system with sensory and motor noise. *J Comput Neurosci*. 2011; 30(3): 759–778. 21161357 10.1007/s10827-010-0291-y PMC3108015

[41] Miall RC, Weir DJ, Wolpert DM, Stein JF. Is the cerebellum a Smith Predictor? *J Mot Behav*. 1993; 25(3): 203–216. 12581990 10.1080/00222895.1993.9942050

[42] Foulkes AJ, Miall RC. Adaptation to visual feedback delays in a human manual tracking task. *Exp Brain Res*. 2000; 131(1): 101–110. 10759175 10.1007/s002219900286

[43] Castro P, Ibitoye R, Ellmers T, Kaski D, Arshad Q, Bronstein AM. Towards an explanation for ‘unexplained’ dizziness in older people. *Age Ageing*. 2024; 53(7): afae137. 38965033 10.1093/ageing/afae137 PMC11223895

[44] Staab JP, Eckhardt-Henn A, Horii A, et al. Diagnostic criteria for persistent postural-perceptual dizziness (PPPD): consensus document of the committee for the classification of vestibular disorders of the Bárány Society. *J Vestib Res*. 2017; 27(4): 191–208. 29036855 10.3233/VES-170622 PMC9249299

[45] Murovec B, Spaniol J, Keshavarz B. Individual factors and vection in younger and older adults: how sex, field dependence, personality, and visual attention do (or do not) affect illusory self-motion. *iPerception*. 2024; 15(4): 20416695241270302. 39139549 10.1177/20416695241270302 PMC11320702

[46] Palmisano S, Allison RS, Teixeira J, Kim J. Differences in virtual and physical head orientation predict sickness during active head-mounted display-based virtual reality. *Virtual Real*. 2023; 27(2): 1293–1313. 36567954 10.1007/s10055-022-00732-5 PMC9761034

[47] Alberts B, Selen LPJ, Medendorp WP. Age-related reweighting of visual and vestibular cues for vertical perception. *J Neurophysiol*. 2019; 121(4): 1279–1288. 30699005 10.1152/jn.00481.2018 PMC6485738

